# Molecular and Morphological Characterization of Two *Clinostomum* (Digenea: Clinostomidae) Species with the First Case of *Clinostomum tilapiae* from Turkey

**DOI:** 10.1007/s11686-024-00955-3

**Published:** 2025-01-14

**Authors:** Mehmet Öztürk, Şinasi Umur

**Affiliations:** Department of Parasitology, Faculty of Veterinary Medicine, Ondokuz Mayıs University, Samsun, Turkey

**Keywords:** Birds, *Clinostomum*, *Cox*1, Parasite, Phylogeny, Trematoda

## Abstract

**Purpose:**

The aim of this study was to investigate the presence of *Clinostomum* species in wild birds in Turkey using morphological and molecular methods.

**Methods:**

51 birds of 18 species from seven orders previously reported as definitive hosts of the *Clinostomum* spp. were collected. Identification of the species was made by morphological characteristics and partial sequence of the *cox*1 gene.

**Results:**

This study concludes that *Ardea alba* and *Ardea cinerea* were infected with *Clinostomum complanatum*, while *Ardea purpurea* was infected with *Clinostomum tilapiae*.

**Conclusion:**

*Clinostomum complanatum* has been reported for the first time in the definitive hosts in Turkey. This study is the first molecular report of *C. tilapiae* in definitive hosts and the first report in Turkey. The present work indicates that *Clinostomum* species in the Afrotropic and Palearctic regions can also be found in Turkey.

**Supplementary Information:**

The online version contains supplementary material available at 10.1007/s11686-024-00955-3.

## Introduction

The trematodes of the genus *Clinostomum* Leidy, 1856 are well-known parasites of fish-eating birds that utilize two intermediate hosts in their life cycle: the first intermediate hosts are freshwater snails, while the second intermediate hosts are freshwater fish, and, rarely, frogs and salamanders [1, 2]. Metacercariae develop in the muscles, body cavities, and gills of the fish, while adult trematodes parasitize the mouth and esophagus of fish-eating hosts [2–4]. The cosmopolitan genus *Clinostomum* comprises over 50 nominal species [2–4]. Among these, only 23 are molecularly characterized (Supplementary Table 1). Due to their yellow colour, the metacercariae are commonly referred to as “*yellow grubs*” and are significant in the fish industry due to the lesions they cause in fish [4]. Species of *Clinostomum* are also of zoonotic significance. Clinical cases can arise in countries where the consumption of raw fish is prevalent due to the ingestion of raw fish contaminated with metacercariae. This situation can lead to laryngitis and pharyngitis, resulting in prominent symptoms in the oral cavity or esophagus [1, 5–7].

**Table 1:** List of bird species examined (locality: Bafra, Samsun, (41◦36′N 36◦05′E), Black Sea Region, Turkey)

Order	Species	If*	Iv*	Cl*
Pelecaniformes	Eurasian bittern (*Botaurus stellaris*)		1	
	Great egret (*Ardea alba*)	1	1	8
	Grey heron (*Ardea cinerea*)	1	1	20
	Purple heron (*Ardea purpurea*)	1	1	6
	Little egret (*Egretta garzetta*)		14	
	Little grebe (*Tachybaptus ruficollis*)		1	
Podicipediformes	Great crested grebe (*Podiceps cristatus*)		3	
Suliformes	Great cormorant (*Phalacrocorax carbo*)		1	
Charadriiformes	Yellow-legged gull (*Larus michahellis*)		9	
	Black-headed gull (*Chroicocephalus ridibundus*)		1	
	Caspian Gull (*Larus cachinnans*)		1	
Ciconiiformes	Black stork (*Ciconia nigra*)		1	
	White stork (*Ciconia ciconia*)		8	
Strigiformes	Tawny owl (*Strix aluco*)		1	
	Long-eared owl (*Asio otus*)		1	
	Barn owl (*Tyto alba*)		2	
Accipitriformes	White-tailed eagle (*Haliaeetus albicilla*)		1	
	Eurasian sparrowhawk (*Accipiter nisus*)		3	
Total	**18 species**	**3**	**51**	**34**

*Iv – investigated; If – infected; Cl – collected parasite

**Table 2:** Comparison of measurements of *Clinostomum complanatum* and *Clinostomum tilapiae* (mm)

	*Clinostomum complanatum*			
Reference	Simsek et al. [36]	Caffara et al. [12]	Caffara et al. [12]	Present study
	Metacercariae	Metacercariae	Adult	Adult
Body size	3.9–6.7 × 1.19–2.13	4.49–7.87 × 1.63–2.43	3.4–6.3 × 1.5–2.7	3.9–5.7 × 1.7–2.0
Body length/width	2.5–3.5	2.20–4.36	–	2.2–2.8
Oral collar	0.61–0.91	0.68–1.03	–	757–876
Oral sucker	0.24–0.31 × 0.26–0.48	0.25–0.33 × 0.28–0.50	0.19–0.57 × 0.32–0.85	0.31–0.49 × 0.36–0.53
Ventral sucker	0.58–0.82 × 0.67–0.89	0.63–0.91 × 0.76–0.95	0.60–0.90 × 0.62–0.90	0.61–0.65 × 0.60–0.68
Distance between suckers	0.82–1.01	0.86–1.11	–	1.0-1.07
Anterior testis	0.29–0.71 × 0.20–0.49	0.31–0.95 × 0.27–0.55	0.55–0.75 × 0.36–0.60	0.37–0.45 × 0.51–0.61
Posterior testis	0.21–0.39 × 0.38–0.56	0.24–0.44 × 0.40–0.60	0.60–0.94 × 0.30–0.51	0.27–0.48 × 0.60–0.83
Distance between testes	0.22–0.44	0.19–0.39	–	0.36–0.53
Cirrus sac	0.21–0.39	0.17–0.34 × 0.11–0.15	0.35–0.40 × 0.10–0.20	0.21–0.30
Ovary	0.12–0.15 × 0.08–0.14	0.13–0.16 × 0.097–0.17	0.22–0.31 × 0.14–0.30	0.14–0.15 × 0.14–0.15
Egg	–	–	0.10–0.125 × 0.065–0.09	0.062–0.125 × 0.041–0.075

	*Clinostomum tilapiae*			
Reference	Caffara et al. [9]	Ukoli [11]	Ukoli [11]	Present study
	Metacercariae	Metacercariae	Adult	Adult
Body size	2.4–7.3 × 0.48–2.2	3.76–5.39 × 1.57–1.71	4.38–7.15 × 1.79–2.42	7.8–7.82 × 2.4–2.41
Body length/width	1.49–3.28	-	-	3.2
Oral sucker	0.14–0.38 × 0.17–0.50	0.26–0.32 × 0.30–0.48	0.26–0.42 × 0.39–0.50	0.20–0.27 × 0.48–0.49
Ventral sucker	0.36–1.14 × 0.36–1.14	0.56–0.80 × 0.70–0.76	0.61–1.07 × 0.65–0.92	0.91–0.93 × 0.85–0.86
Distance between suckers	0.24–1.64	0.48–0.70	0.52–1.07	1.2–1.22
Anterior testis	0.19–0.53 × 0.23–0.73	0.22–0.33 × 0.30–0.37	0.48–0.67 × 0.61–0.78	0.45–0.46 × 1.01–1.02
Posterior testis	0.22–0.58 × 0.21–0.83	0.22–0.32 × 0.32–0.47	0.39–0.64 × 0.70–0.92	0.44 × 0.8–0.83
Distance between testes	0.24–0.62	0.16–0.19	0.13–0.21	0.19–0.20
Cirrus sac	0.24–0.35 × 0.14–0.33	0.22–0.26 × 0.10–0.15	0.30–0.35 × 0.21–0.24	0.35–0.39 × 0.27–0.29
Ovary	0.13–0.21 × 0.07–0.12	0.08–0.11 × 0.04–0.07	0.17–0.21 × 0.13–0.14	0.14–0.15 × 0.14–0.15
Egg	–	–	–	0.089–0.103 × 0.064–0.066

The members of the genus *Clinostomum* exhibit high interspecific morphological similarities but also show intraspecific variability. Therefore, it is crucial to integrate molecular data with morphological observations to achieve accurate taxonomic resolutions. Taxonomic revisions within this genus are most effectively carried out by combining morphological and molecular approaches. This integrative approach allows for a more comprehensive understanding of species diversity and relationships within the genus *Clinostomum*.

## Material & Methods

### Trematode Collection and Morphological Studies

In this study, the presence of *Clinostomum* spp. was investigated in wild birds that were brought to the Department of Wild Animal Diseases at Ondokuz Mayıs University, Faculty of Veterinary Medicine, and which did not recover after treatment, as well as in wild birds found dead in the Kızılırmak Delta area in the Bafra district of Samsun Province, Turkey, in 2023. This investigation was conducted with permission dated September 26, 2022, and numbered E-21264211-288.04-7092178, obtained from the Ministry of Agriculture and Forestry, General Directorate of National Parks.

A total of 51 birds of 18 species from seven orders, previously reported as definitive hosts, were collected (Table 1) [8]. These wild birds had died naturally in the Kızılırmak Delta. They were then transported to the laboratory for a necropsy. As a result of the parasitological examination, several helminths, including specimens of *Clinostomum* spp., were obtained. These were washed in 0.9% saline and preserved in 70% ethanol for morphological identification. The posterior end of the collected *Clinostomum* trematodes was removed and stored in 70% ethanol for molecular study [9]. Permanent mounts were prepared using Semichon’s carmine following the standard procedure, and then they were mounted with Canada balsam for morphological analysis [10]. The specimens were identified using previously reported morphological features under a light microscope (Nikon Eclipse 80i) [9, 11, 12]. The special morphological characters were measured and photographed using the camera (Nikon DS-L1) integrated into the microscope, and illustrations were created with Adobe Illustrator 2020.

### Molecular Studies

According to the manufacturer’s instructions, genomic DNA (gDNA) extractions of the parasites were performed using the GeneJet Genomic DNA purification kit (Thermo Scientific, USA). Genomic DNA extracts were stored at -20 °C until molecular analyses were performed. *Clinostomum* species were molecularly characterised by PCR using Dice1F (5′- ATTAACCCTCACTAAATTWCNTTRGATCATAAG-3) as the forward primer and Dice11R (5-TAATACGACTCACTATAGCWGWACHAAATTTHCGATC-3) as the reverse primer, in partial cytochrome *c* oxidase subunit 1 gene (*cox*1) region [13]. The commercial master mix was adjusted to a 25 µl PCR reaction mix (Maxima Hot Start PCR Master Mix, Thermo Scientific, Waltham, MA, USA), containing 10 µM of each primer, 2 µl genomic DNA (25–50 ng of gDNA) and nuclease-free water to a total volume of 50 µl. Amplification conditions were programmed as follows: initial denaturation 95 °C for 10 min, 40 cycles of denaturation at 95 °C for 45 s, annealling at 55 °C for 45 s, elongation at 72 °C for 45 s and final elongation at 72 °C for 5 min [13].

A commercial company (Bm Laboratory Systems, Turkey) was used for purification and sequencing. The same primers were used with the ABI PRISM 3130xl automated sequencer (Applied Biosystems) along with the BigDye Terminator v3.1 Cycle Sequencing kit provided by Macrogen (Amsterdam, The Netherlands). The quality scores of the chromatograms showing the base sequences of the bidirectional DNA sequences were analyzed using the Vector NTI Advance 11.5 (Invitrogen) genetic software program. Subsequently, the forward and reverse sequences were assembled using Contig Express in Vector NTI Advance 11.5 (Invitrogen), and a consensus nucleotide sequence for each isolate was obtained. The obtained sequences were analyzed using BLASTn (http://blast.ncbi.nlm.nih.gov/Blast.cgi) in the GenBank database to determine the homologies and similarity percentages of the isolates with each other and with other isolates worldwide [14]. Multiple alignments of nucleotide sequences were conducted using the Clustal W algorithm with the BioEdit program [15], trimmed to the shortest sequence length, manually refined using BioEdit, and saved in FASTA format. The haplotype groups of the isolates were identified using the DnaSP v6.10 program [16]. The genetic distances of the isolates (pairwise distance, K2P) were determined using the MEGA software 11.0.13 [17]. To investigate phylogenetic relationships, Bayesian inference (BI) analysis was conducted using MrBayes version 3.2.3 [18]. jModelTest version 2.1.6 was used to find the most suitable DNA model for the generated data sets [19]. *Ithyoclinostomum dimorphum* Diesing, 1850 (Clinostomidae: Diplostomida) (OP174428) was used as an outgroup in BI trees. The trees were visualized using FigTree ver. 1.4.4 software (http://tree.bio.ed.ac.uk/software/figtree/).

## Result

### Morphological Results

**Fig. 1:**
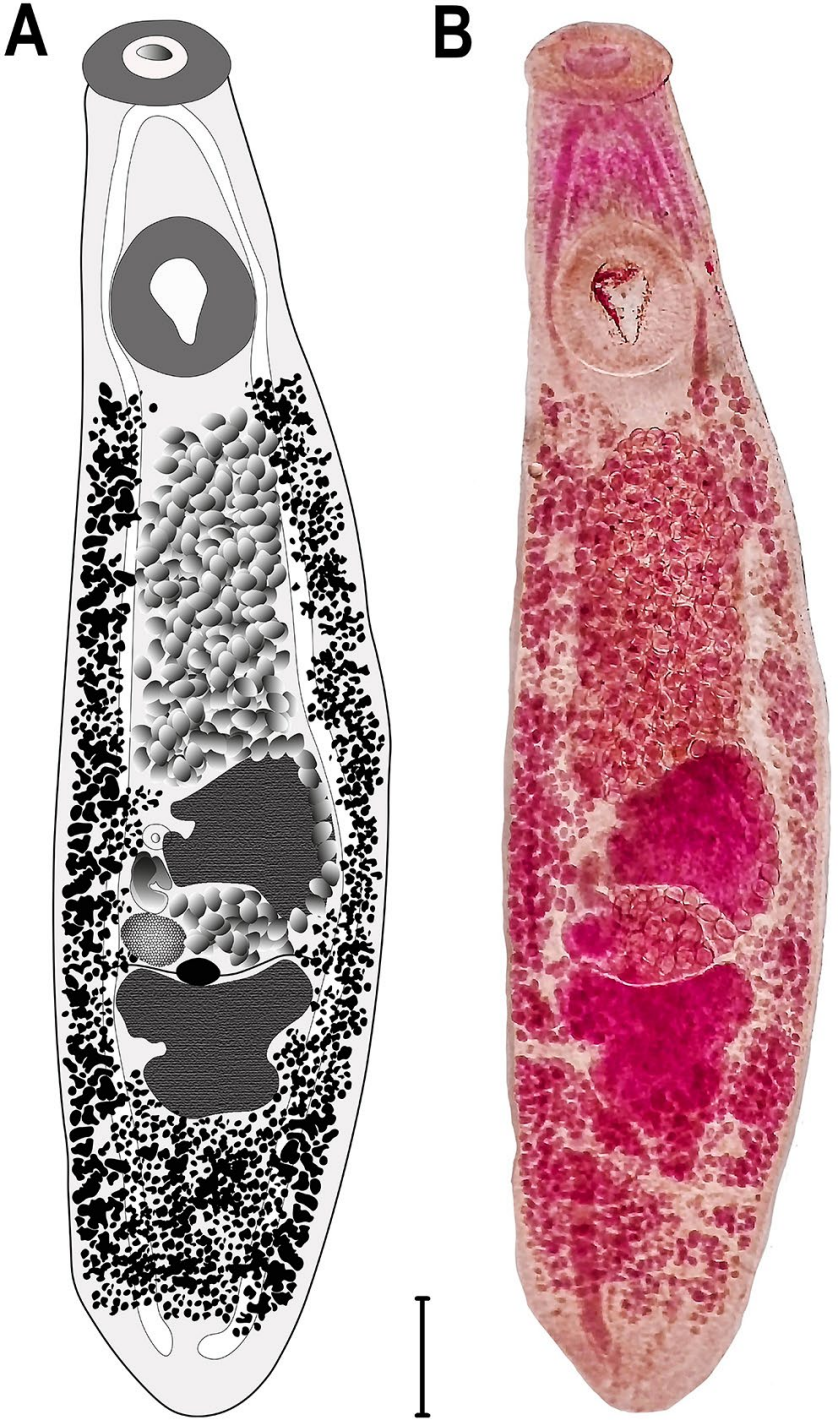
*Clinostomum complanatum*, **a**) Illustration, **b**) Photomicrograph. Scale b= 1000 μm

**Fig. 2:**
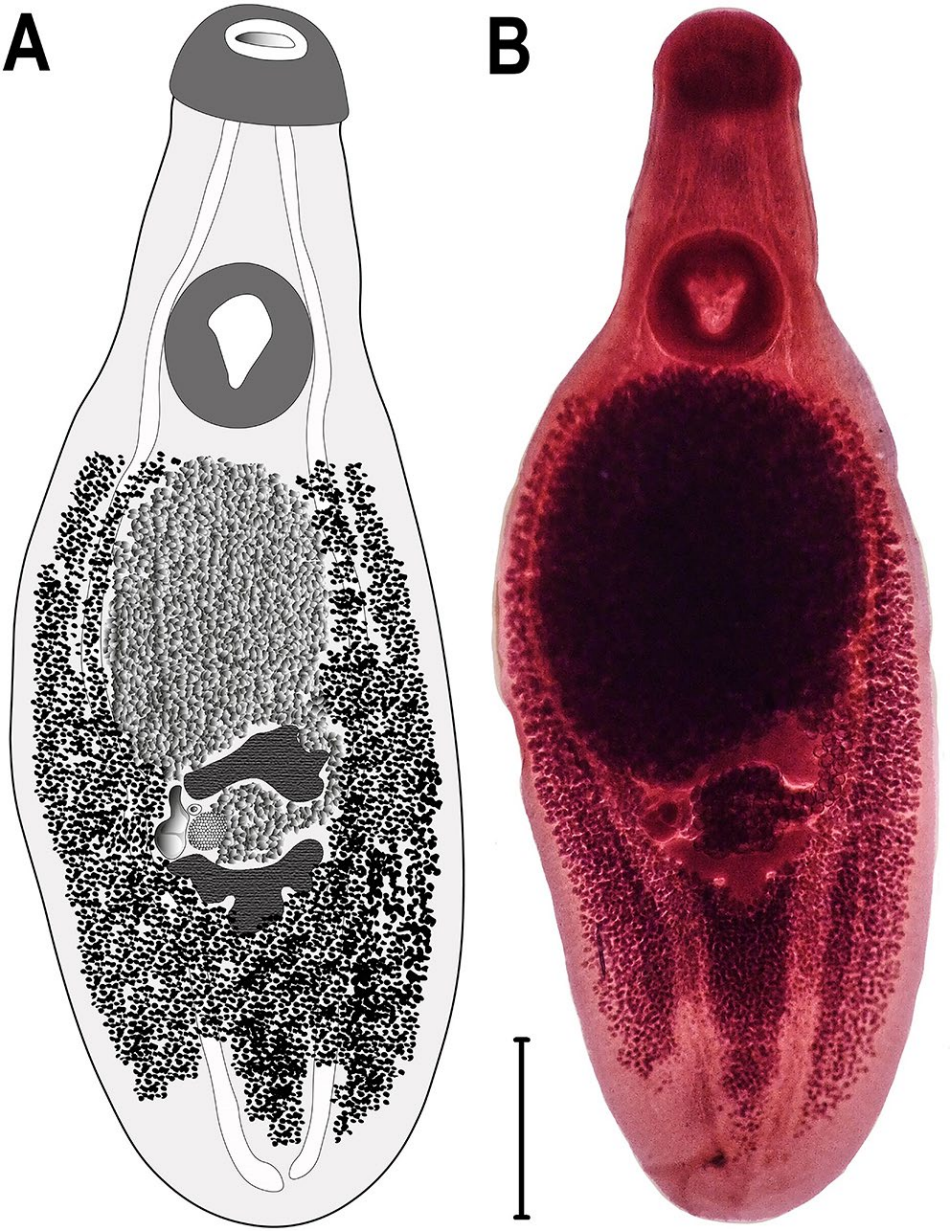
*Clinostomum tilapiae*, **a**) Illustration, **b**) Photomicrograph. Scale bar = 1000 μm

In total, only 3 of the 51 birds (5.88%) were infected with *Clinostomum* spp. One *Ardea alba* and one *A. cinerea* were infected with 8 and 20 specimens of *Clinostomum complanatum*, respectively, while one *A. purpurea* was infected with six specimens of *C. tilapiae* Fig. 1 and Fig. 2. Identification was performed both morphologically and molecularly by studying the *cox*1 gene region, guided by relevant literature [9, 11–13].

#### *Clinostomum complanatum* (Rudolphi, 1814) Braun, 1899

Host: *Ardea alba* Linnaeus, 1758, *Ardea cinerea* Linnaeus, 1758.

Site of infection: Oral cavity, oesophagus.

Intensity of infection: 8, 20.

Locality: Bafra, Samsun, (41°36′N 36°05′E), Black Sea Region, Turkey.

Material deposited: Specimens were deposited in the Helminth Coll. No. OMUPAR.873.23.01- OMUPAR.874.23.01 Department of Parasitology, Faculty of Veterinary Medicine, Ondokuz Mayıs University, Samsun, Turkey.

Adult (based on five specimens): Body thick, elongated, tapering from posterior sucker towards anterior end, 3.9–5.7 long by 1.7–2.0 mm wide. Length-to-width ratio of 2.2 to 2.8. Ventral side convex, and tegument spined. Oral sucker subterminal, round, 317–490 × 366–530 μm. Oral collar present, 757–876 μm. Ventral sucker located at posterior end of anterior third of body, oval, 615–650 × 607–688 μm. Ventral sucker width/oral sucker width 0.51–0.75. Cecum extends to posterior end of body and has irregular serrations. Prepharynx or pharynx not present. Testes lobed, tandem, and triangular, locates very close to each other in posterior part of body. Anterior testis 370–450 × 510–610 μm, and posterior testis 276–488 × 608–830 μm. Cirrus aligns with ovary on right side between testes. Genital pore located in front of anterior testis. Ovary ovoid, intertesticular, to left of cirrus, 144–150 × 148–152 μm. Vitelline glands occupy entire middle third of body and anterior part of posterior third. Uterus curves from left of anterior testis, join uterine sac. Uterine sac fills entire area between anterior testis and ventral sucker. Eggs measure 62–125 × 41–75 μm (based on five eggs).

#### Clinostomum tilapiae Ukoli, 1966

Host: *Ardea purpurea* Linnaeus, 1766.

Site of infection: Oral cavity.

Intensity of infection: 6.

Locality: Bafra, Samsun, (41°36′N 36°05′E), Black Sea Region, Turkey.

Material deposited: Specimens were deposited in the Helminth Coll. No. OMUPAR.875.23.01 Department of Parasitology, Faculty of Veterinary Medicine, Ondokuz Mayıs University, Samsun, Turkey.

Adult (based on two specimens): Body thick, elongated, tapers from posterior sucker towards anterior end, 7.8–7.82 mm long by 2.4–2.41 mm wide. Length-to-width ratio of 3.2. Ventral side convex, tegument spined. Oral sucker subterminal, round, 207–227 × 480–492 μm. Ventral sucker locates at posterior end of anterior third of body, oval, 917–937 × 858–866 μm. Ventral sucker width/oral sucker width 0.23–0.25 μm. Cecum extends to posterior end of body and has irregular serrations. Prepharynx or pharynx not present. Testes lobed, tandem, triangular, and locates very close to each other in posterior part of body. Anterior testis 454–466 × 1014–1022 μm, and posterior testis 444–448 × 808–832 μm. Cirrus aligns with ovary on right side between testes. Genital pore locates in front of anterior testis. Ovary ovoid, intertesticular, to left of cirrus, 144–150 × 148–152 μm. Vitelline glands occupy entire middle third of body and anterior part of posterior third. Uterus curves from left of anterior testis, join uterine sac. Uterine sac fills entire area between anterior testis and ventral sucker. Eggs measure 89–103 × 64–66 μm (based on five eggs).

### Molecular Results

Three partial sequences of *cox*1 were obtained: two from *C*. *complanatum* isolated from *Ardea alba* and *A.cinerea* (511 and 613 bp, respectively; differences of 0.01%) and one from *C. tilapiae* isolated from *A. purpurea* (604 bp), respectively. All *Clinostomum* isolates (45 isolates) that matched our data according to BLASTn search were included in the study. Haplotype analysis of *C. complanatum* isolates (30 isolates) from different geographical regions included in the study and our isolates was performed with the DnaSP v6.10 program, identifying 16 haplotypes. The intraspecific genetic distance between partial *cox*1 sequences of *C. complanatum* ranged from 0.01 to 0.03%.

Our sample from *A. alba* (PP833143) shared 100% identity with the published *C. complanatum* metacercariae sequences (OP678025 and MK801718) from *Capoeta capoeta* in Iran and *Scardinius erythrophthalmus* in Romania. Our second sample from *A. cinarea* (PP833144) shared 99.61% identity with the published *C. complanatum* metacercariae sequence (MK801718) from *S. erythrophthalmus* in Romania [20].

Our *C. tilapiae* sample from *A. purpurea* matched with other isolates (nine isolates) according to BLASTn search. Haplotype analysis of *C. tilapiae* isolates (nine isolates) from different geographical regions, including our isolate, was performed with the DnaSP v6.10 program, identifying four haplotypes. The intraspecific genetic distance between *cox*1 sequences of *C. tilapiae* ranged from 0.003 to 0.01%. Our *C. tilapiae* isolate (PP833145) haplotype shared 99.83% identity with the published *C. tilapiae* metacercariae sequence (KY649361) from *Synodontis batensoda* in Nigeria [9].

**Fig. 3:**
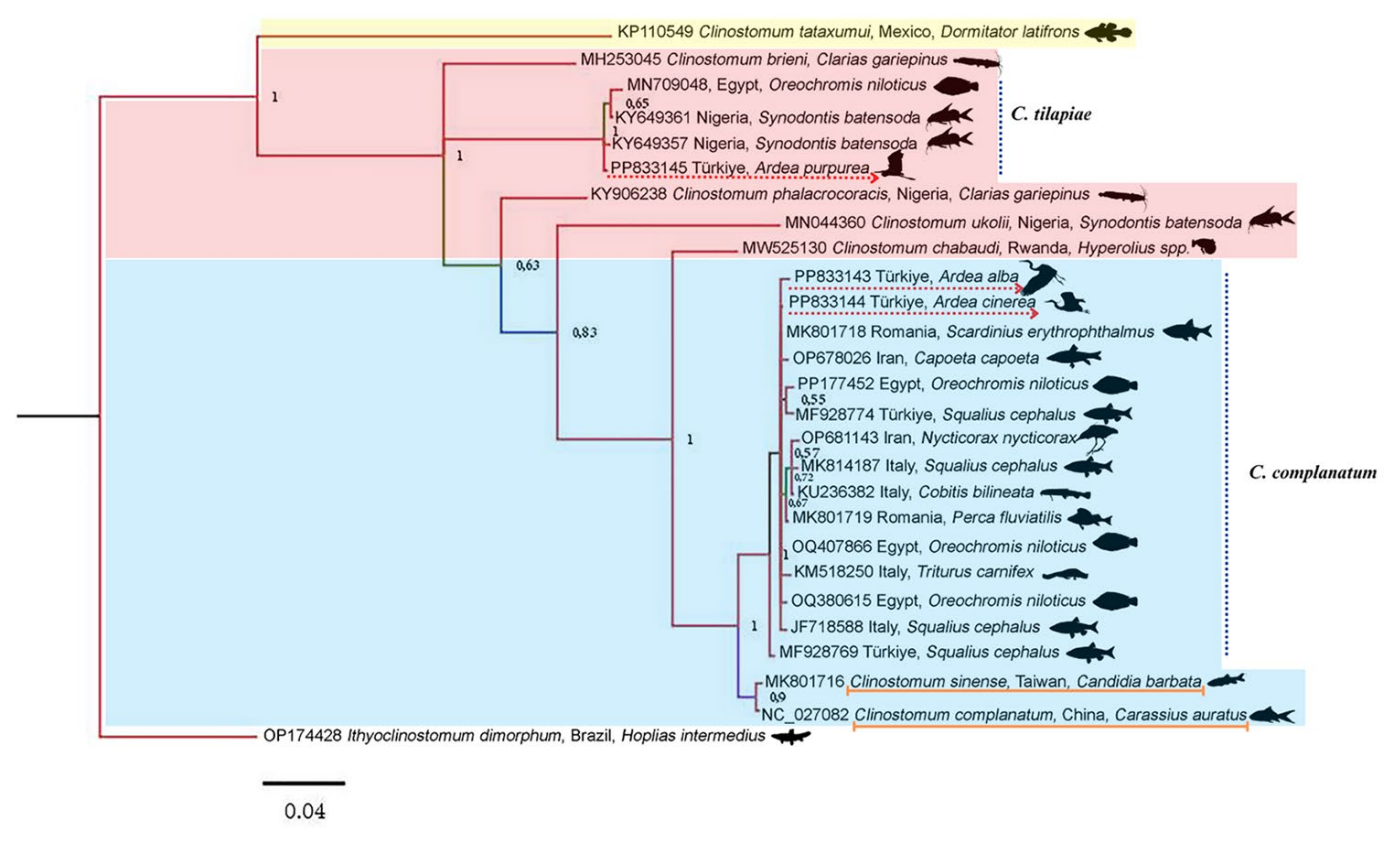
The Bayesian inference (BI) tree was constructed based on the cytochrome *c* oxidase subunit 1 gene dataset. *Ithyoclinostomum dimorphum* (OP174428) was selected as an outgroup. The BI tree was generated using the HKY + I + G model. The appearance was colored with the prop option in the Figtree program. The phylogenetic tree of *cox*1 sequences revealed three distinct clades: the Afrotropical biogeographic region (pink), the Palearctic biogeographic region (blue), and the Nearctic/Neotropical biogeographic region (yellow). The *C. sinense* isolate obtained from *Carassius auratus* is mislabeled as *C. complanatum*, and the *C. sinese* isolate is represented by the orange line. The scale bar indicates the average number of substitutions per site

The most optimal DNA model for the generated data set was identified as HKY + I + G. The phylogenetic tree of *cox*1 sequences revealed three distinct clades: one from the Afrotropical biogeographic region (*C. brieni*, *C. chabaudi*, *C. phalacrocoracis*, *C. tilapiae*, *C. ukoili*), one from the Palearctic biogeographic region (*C. complanatum*, *C. sinense*), and one from the Nearctic/Neotropical biogeographic regions (*C. tataxumui*) (Fig. 3). The obtained sequence data were recorded in GenBank under the accession numbers PP833143 – PP833145.

**Fig. 4:**
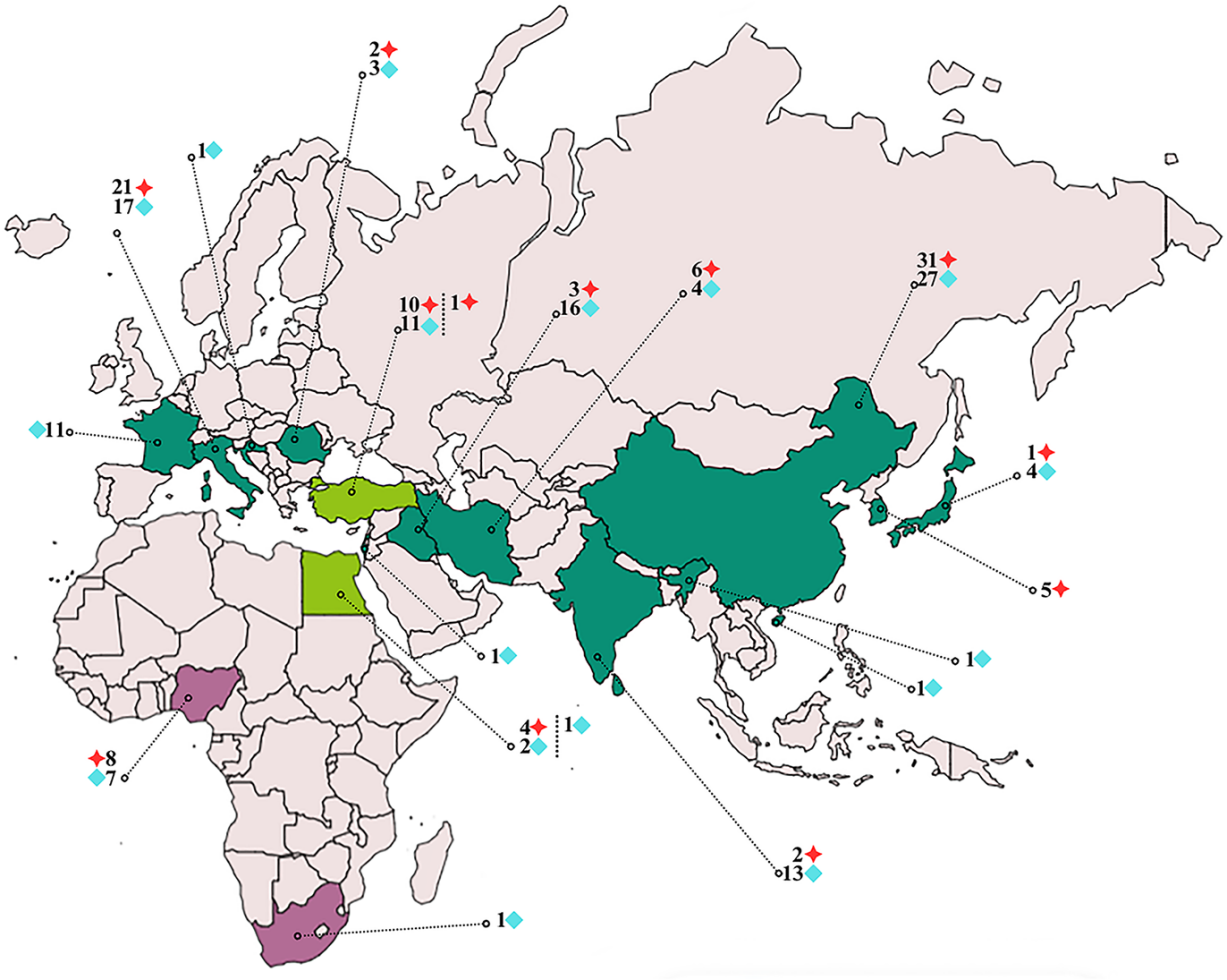
According to molecular data, the distribution of *C. complanatum* (dark green) and *C. tilapiae* (purple) species is presented. The red marker indicates studies in the mitochondrial DNA (*mtDNA*) gene region, and the light blue marker indicates studies in the ribosomal RNA (*rRNA*) gene region. In countries where the two species are co-occurr (light green), studies on *C. complanatum* are indicated before the dashed line and *C. tilapiae* after the dashed line. Data were retrieved from GenBank (https://www.ncbi.nlm.nih.gov/)

## Discussion

*Clinostomum* species are cosmopolitan parasites of significant zoonotic importance. Examinations conducted in the Nearctic and Neotropic biogeographic regions have molecularly documented species such as *C. album*, *C. arquus*, *C. caffarae*, *C. cichlidorum*, *C. dolichorchum*, *C. marginatum*, and *C. tataxumui* [21–23]. In the Afrotropic biogeographic regions, species such as *C. brieni*, *C. chabaudi*, *C. cutaneum*, *C. phalacrocoracis*, *C. tilapiae*, and *C. ukoili* have been molecularly reported [9, 24, 25]. In the Palearctic biogeographic region, species such as *C. complanatum*,* C. giganticum*,* C. heterostomum*, and *C. sinense* have been reported [26].

*Clinostomum complanatum* was first described in 1819 by Rudolphi in Berlin as *Distoma complanatum* [27]. It is a typical European species reported by Braun [28] in *Ardea cinerea* from Germany, and as metacercariae by Grabda-Kazubska [29] from *Perca fluviatilis* and *Rutilus rutilus* in Poland and by Vilizzi et al. [30] from *Cyprinus carpio* in Italy. Outside Europe, *C*. *complanatum* was also reported from *A*. *cinerea* in Iraq [31], from *Microcarbo niger* in Pakistan [32], and from *Lymnaea gedrosian* (first intermediate host) in Iran [33–34]. Metacercariae were reported by Malek and Mobedi [35] from *Capoeta capoeta* in Iran, while Şimşek et al. [36] reported metacercariae in the fish *Squalius cephalus* including molecular data. Caffara et al. [12, 21] reported adults from *A*. *cinerea*, *A*. *purpurea*, and *Egretta garzetta* and metacercariae from *Barbus barbus*, *Lissotriton vulgaris*, and *Triturus carnifex* in Italy.

Chen et al. [37] conducted a complete mitochondrial genome analysis of *C. complanatum* from *Carassius auratus* collected in Hubei, China. However, a study by Locke et al. [20] demonstrated that this species is not *C. complanatum* but a new species, *C. sinense*, which is also supported by the present study (Fig. 3). Furthermore, based on molecular analyses and GenBank sequences, we believe that *C. complanatum* is not only a European species but is also widespread in the western Palearctic region (Fig. 4).

*Clinostomum tilapiae* was first described by Ukoli in 1966 from metacercariae found in fish (*Tilapia* spp.) from Ghana, and from experimentally obtained adults in the cattle egret (*Bubulcus ibis*) [11]. Later, Manter and Pritchard [38] identified adult *C*. *tilapiae* in *Ardea goliath* from Lake Kisale, Democratic Republic of the Congo, Fischthal and Thomas [39] reported metacercariae in *Tilapia zilli*, *T*. *heudeloti*, and *T*. *galilaea* in Ghana. Britz et al. [40] identified adults in *A*. *cinerea* and metacercariae in *O*. *mossambicus* in South Africa. Finkelman et al. [41] documented adults in *Pelecanus onocrotalus* and metacercariae in *Sarotherodon galilaeus* and *Oreochromis aureus* in Lake Kinneret, Israel [41, 48]. Metacercariae have also been confirmed in *Chromidotilapia guntheri*, *T*. *mariae*, *T*. *zilli*, *Hemichromis fasciatus*, and *O*. *niloticus* in Nigeria [42–47], in Cyprinus carpio in Mozambique [49], and fin *O*. *niloticus* in Egypt [50].

Ukoli [11] demonstrated that the position of the testes changes according to the developmental stage of *C. tilapiae* rather than its body length, and that the largest parasite is not necessarily the oldest. The study revealed that the parasite’s body measurements reached their maximum length and width within two days, and as the developmental period progressed, the body length, testes, and ventral sucker decreased in size, while the length and width of the ovary increased. It was also noted that other organs remain relatively stable in metacercariae and adults. Therefore, since the age of the parasites obtained from naturally infected birds is unknown, the morphological differences and size variations indicate that these factors depend on the age of the parasite and the host species. The *C. tilapiae* specimens collected in this study were slightly larger than the metacercariae and adult parasites reported by Ukoli (1966) and the metacercariae described by Caffara (2017) (Table 2). On the other hand, the *C. complanatum* specimens reported in this study are consistent with previously reported morphometric measurements. The genital complex is located in the middle one-third of the body in *C. tilapiae*, with the posterior testis located at the border of the posterior one-third of the body; in *C. complanatum*, it is located between the middle and posterior one-third of the body. The anterior testis in *C. tilapiae* is irregularly lobed and longitudinally asymmetrical, while the posterior testis has three lobes. In *C. complanatum*, the anterior testis is displaced to the left by the cirrus. The cirrus sac is oval between the testes in *C. tilapiae* and extends from the intertesticular space to the right margin of the anterior testis in *C. complanatum* [12, 24].

Our results show that *Clinostomum* species, which are found in both the Afrotropic and Palearctic regions, can also occur in Turkey (Fig. 4). Turkey’s geographic location places it on the natural migration routes of birds. Fish-eating birds, which serve as the final hosts for *Clinostomum* species, breed, reproduce, and spend their summers or winters in Turkey. These activities contribute to the parasites’ distribution across ecological regions through their final hosts. Combining morphological and molecular data with environmental and geographical insights will significantly enhance the understanding of the epidemiology and control strategies for *Clinostomum* species.

## Electronic Supplementary Material

Below is the link to the electronic supplementary material.

Supplementary Material 1

## Data Availability

Data available on GenBank accession numbers PP833143-PP833145 and museum voucher OMUPAR.873.23.01- OMUPAR.874.23.01 and OMUPAR.875.23.01.
