# Supplementary material for: Molecular and Morphological Characterization of Two Clinostomum (Digenea: Clinostomidae) Species with the First Case of Clinostomum tilapiae from Turkey
Source: Acta Parasitol. 2025 Jan 14;70(1):18. doi: 10.1007/s11686-024-00955-3 (PMC11732774; doi:10.1007/s11686-024-00955-3)
Supplement: Supplementary file 1 — Supplementary Material 1 [file 11686_2024_955_MOESM1_ESM.docx]

| **Table 1.** List of specimens used for phylogenetic analysis. | | |  |  |
| --- | --- | --- | --- | --- |
| **Species** | **Host** | **Location** | **Accesion number** | **Reference** |
| *C. album* | *Planorbella trivolvis* | Mississippi, USA | MH282542 | Rosser et al. 2018 |
| *C. arquus* | *Pseudoxiphophorus jonesii* | Presa Los Ocotes, Mexico | KJ477565 | Pérez-Ponce de León et al. 2016 |
| *C. attenuatum* | *Lithobates sp.* | Quebec, Canada | KP150305 | Locke et al. 2015 |
| *C. brieni* | *Clarias gariepinus* | Limpopo, South Africa | MH253044 | Caffara et al. 2019 |
| *C. caffarae* | *Egretta thula* | Lago de Catemaco, Mexico | KU156797 | Pérez-Ponce de León et al. 2016 |
| *C. chabaudi* | *Hyperolius spp.* | Muhanga, Rwanda | MW525130 | Sinsch et al. 2021 |
| *C. cichlidorum* | *Archocentrus siquia* | Rio Las Vueltas, Costa Rica | KU156816 | Pérez-Ponce de León et al. 2016 |
| *C. complanatum* | *Triturus carnifex,*  *Lissotriton vulgaris* | Italy | KM518246 | Caffara et al. 2014 |
| *C. cutaneum* | *Ardea cinerea* | Central, Kenya | KP110515 | Locke et al. 2015 |
| *C. detruncatum* | *Synbranchus marmoratus* | Guaira, Brazil | KP110518 |  |
| *C. dolichorchum* | *Ardea herodias*  *Rana catesbeiana* | Arkansas, USA | PP411897 | Nguyen et al. 2024 |
| *C. giganticum* | *Channa punctatus* | India | KY312846 |  |
| *C. fergalliarii* | *Ardea cocoi* | Magdalena, Buenos Aires, Argentina | MW187309 | Montes, et al. 2021 |
| *C. heluans* | *Ardea alba*  *Australoheros sp.* | Mexico  Brazil | MG860853  MG860852 | Briosio-Aguilar et al. 2018 |
| *C. marginatum* | *Rana clamitans*  *Lepomis gibbosus*  *Lepomis macrochirus*  *Catostomus nebuliferus*  *Ardea alba* | Quebec, Canada  Mississippi, USA  Durango, Mexico  Veracruz, Mexico | JF718618 JF718619  MH282538  JX630993  JX630995 | Caffara et al. 2011  Rosser et al. 2018  Sereno-Uribe et al. 2013 |
| *C. phalacrocoracis* | *Ardea cinerea* | Central, Kenya | KP110522 | Locke et al. (2015) |
| *C. philippinense* | *Trichogaster microlepis* | Thailand | KP110523 |  |
| *C. piscidium* | *Trichogaster fasciata* | India | KY290512 | Choudhary et al. 2022 |
| *C. poteae* | *Phalacrocorax auritus* | Mississippi, USA | MH282550 | Rosser et al. 2018 |
| *C. sinense* | *Candidia barbata* | Taiwan | MK801713 | Caffara et al. 2019 |
| *C. tataxumui* | *Tigrisoma mexicanum* | Laguna Manialtepec, Mexico | KJ504192 | Pérez-Ponce de León et al. 2016 |
| *C. tilapiae* | *Synodontis batensoda* | Anambra River, Nigeria | KY649357 | Caffara et al. 2017 |
| *C. ukolii* |  |  | MN044350 | Caffara et al. 2020 |
